# Postnatal clinical phenotype of five patients with Pallister–Killian Syndrome (tetrasomy 12p): Interest of array CGH for diagnosis and review of the literature

**DOI:** 10.1002/mgg3.939

**Published:** 2019-08-27

**Authors:** Amerh Salem Alqahtani, Audrey Putoux, Marie Noelle Bonnet Dupeyron, Maryline Carneiro, Laurence Lion‐Francois, Massimiliano Rossi, Hélène Tevissen, Caroline Schluth Bolard, Audrey Labalme, Gaetan Lesca, Marianne Till, Patrick Edery, Damien Sanlaville

**Affiliations:** ^1^ Department of Medical Genetics, Women Mothers and Children's Hospital Lyon Hospices Civils Lyon France; ^2^ Ministry of Higher Education Riyadh Saudi Arabia; ^3^ Claude Bernard Lyon 1 University Lyon France; ^4^ CRNL, CNRS UMR 5292 INSERM U1028 Lyon France; ^5^ Department of Genetics Valence Hospital's Center Valence France; ^6^ Department of Neuropediatrics, Women Mothers and Children's Hospital Lyon Hospices Civils Lyon France; ^7^ Department of Pediatrics Valence Hospital's Center Valence France

**Keywords:** aCGH, isochromosome 12p, Pallister–Killian syndrome, postnatal, tetrasomy 12p

## Abstract

**Background:**

Pallister–Killian syndrome (PKS) is a rare sporadic disorder caused by tetrasomy of the short arm of chromosome 12. The main clinical manifestations are global developmental delay, intellectual disability, epilepsy, dysmorphic features, hypopigmented and/or hyperpigmented lesions, and multiple congenital anomalies. PKS is associated with tissue mosaicism, which is difficult to diagnose through peripheral blood sample by conventional cytogenetic methods and *fluorescence* in situ *hybridization*.

**Methods:**

Here, we report five patients with PKS. We delineate their clinical phenotypes and we compare them with previously published cases. We used array Comparative Genomic Hybridization (aCGH) with DNA extracted from peripheral blood samples. The five patients have also been tested by conventional cytogenetics techniques.

**Results:**

Four out of five patients showed tetrasomy 12p by aCGH. Three of the four patients have typical i(12p) and one of the four demonstrated atypical tetrasomy 12p. The percentage of mosaicism was as low as 20%. Our cohort exhibited the typical PKS phenotypes.

**Conclusion:**

Our results demonstrate the efficacy of aCGH for the diagnosis of PKS from DNA extracted from lymphocytes. Thus, for patients suspected of PKS, we recommend performing aCGH on lymphocytes at an early age before  proceeding to skin biopsy. aCGH on peripheral blood samples is sensitive in detecting low level of mosaicism and it is less invasive method than skin biopsy. We reviewed also the literature concerning the previously published PKS patients diagnosed by aCGH.

## 1 INTRODUCTION

Pallister–Killian syndrome (OMIM 601803; PKS) is a rare disorder which was first described by Philip Pallister (Pallister et al., 1977). PKS is characterized by intellectual disability (ID), multiple congenital anomalies, streaks of hypo‐ and/or hyperpigmentation, and dysmorphic features including a high forehead with frontotemporal alopecia, upslanting palpebral fissures with epicanthal folds, hypertelorism, flat nasal bridge, short nose with upturned nares, low‐set ears, macrostomia, eversion of lower lip and thin upper lip with invasion of vermilion border of upper lip by philtral skin, which is known as “Pallister lip”, short neck and macrosomia at birth (Blyth et al., 2015; Karaman et al., 2018; Wilkens et al., 2012). Supernumerary nipples may also be found. Patients generally show developmental delay, axial hypotonia, profound ID, epilepsy, and deafness. Several congenital malformations may be present including congenital heart and lung defects, gastrointestinal disorders like congenital diaphragmatic hernia (CDH), anal atresia, renal anomalies, and cryptorchidism (Blyth et al., 2015; Karaman et al., 2018; Wilkens et al., 2012). PKS is caused by the presence of a supernumerary isochromosome i(12p), in mosaic status. This extra chromosome can generally be detected on cultured fibroblast by karyotype or FISH, but the detection of i(12p) might be complicated on cultured lymphocyte. In the last decade, several publications show that array Comparative Genomic Hybridization (aCGH) on DNA extracted from lymphocytes could identify tetrasomy 12p at mosaic level (Blyth et al., 2015; Chen et al., 2014; Lee, Lee, Yu, Lee, & Kim, 2017; Theisen et al., 2009).

Here, we diagnosed four out of five PKS patients using the aCGH on blood‐extracted DNA and illustrate their cytogenetics results. Additionally, we delineate their clinical phenotypes and we compare them with previously published patients.

## 2 MATERIALS AND METHODS

### 2.1 Ethical compliance

This study has been approved by the ethics committee.

### 2.2 Clinical reports

This is a descriptive study based on the medical records of five patients with confirmed PKS. The five patients were referred to the genetics department in Women Mothers and Children's hospital in Lyon, France. Four boys and one girl aged from 3 to 11 years old were included. All of them displayed moderate/severe global developmental delay and typical facial dysmorphic features corresponding to PKS. Patient 1 was a 4 and half year‐old boy presented with moderate developmental delay. Patient 2 was a 7‐year‐old boy who exhibited severe developmental delay, macrosomia at birth, and epilepsy starting at 7 years old. Patient 3 was a 3‐year‐old girl who was diagnosed at birth. She showed axial hypotonia, facial dysmorphism, and umbilical hernia. Patient 4 was a 9‐year‐old boy. The antenatal history was notable for polyhydramnios. He demonstrated severe developmental delay, macrosomia at birth, and epilepsy starting at 7 and half years old. Patient 5 was a 11‐year‐old boy. The pregnancy was notable for polyhydramnios. He showed developmental delay and epilepsy starting at 7 years old (Table 2).

### 2.3 Conventional cytogenetic analysis

Karyotype studies were performed, on all patients, using a standard phytohemagglutinin‐stimulated lymphocyte culture method, followed by G‐and R‐banding techniques. Skin biopsy was subjected to fibroblasts culture according to standard protocol for patients 4 and 5.

### 2.4 FISH

Subtelomeric chromosome 12p specific probe (12ptel27) and chromosome 12 centromere specific probe (D12Z3) were used for FISH studies (Cytocell). Additionally, 12q12 specific probe (PR11‐478B9) was used for patient 3. Samples were taken from blood for patients 1, 2, 3, and 5 and from skin biopsy for patients 4 and 5.

### 2.5 aCGH

Genomic DNA was isolated from peripheral blood leukocytes for all patients using a nucleospin blood L kit (Macherey‐Nagel GmbH & Co. KG), according to the manufacturer's protocol. The whole genomic aCGH procedure was performed following the manufacturer's instructions (Sure Print G3 Human CGH Microarray Kit; Agilent Technologies). The 180K slides were scanned on an Agilent DNA Microarray Scanner and images were extracted with Feature Extraction software (12.0.1.1). Data analysis was carried out with Cytogenomics v3.0.3.3. Before 2014, the Feature Extraction software used was the version (11.5.11) and the data interpretation analysis used Workbench v3.4.2.7. The following parameters were used for interpretation: ADM‐2, threshold: 5.0, window: 0.2 Mb, cutoff: 0.25. A copy number variation was validated if an abnormal log2 ratio was obtained for at least three contiguous oligonucleotides. The aCGH results were analyzed with the UCSC hg19 assembly.

## 3 RESULTS

Pallister–Killian syndrome was confirmed in the five patients. We used aCGH on peripheral blood sample in all patients, karyotyping and FISH on peripheral blood sample only in three patients (patient 1, 2 and 3), on skin biopsy only in one patient (patient 4), and on both in one patient (patient 5). The combined results from aCGH and conventional cytogenetic techniques demonstrated the presence of typical i(12p) in patients 1, 2, 4, and 5 and partial tetrasomy of distal 12p (12p13.33p12.1) and partial trisomy of proximal part of 12p and 12q (12p12.1q12) for patient 3.

**Figure 1:**
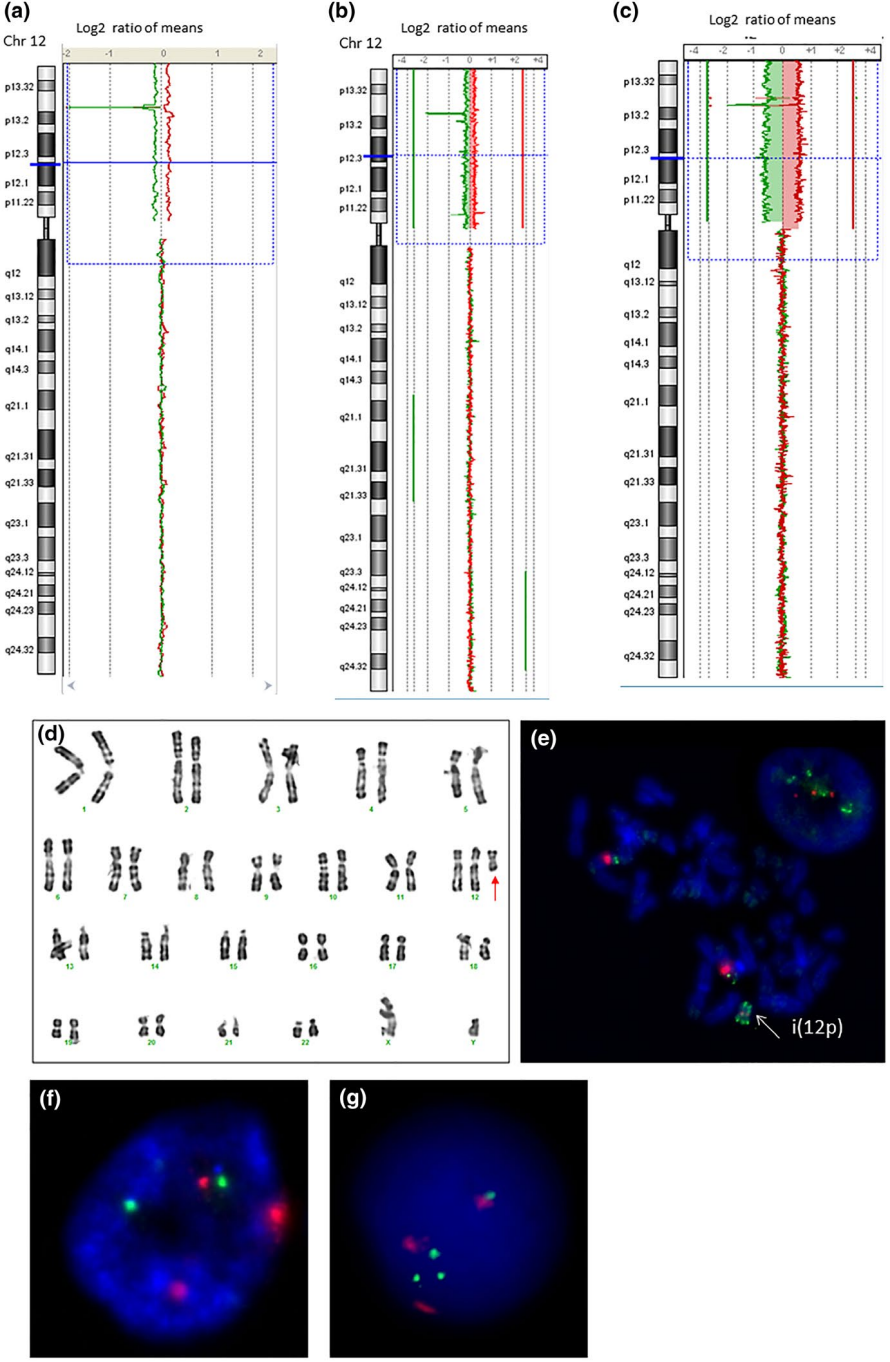
Cytogenetic results for patients 1, 2, and 5. (a‐c) aCGH profiles of uncultured lymphocytes of patients 1 (a), 2 (b), and 5 (c) showing gain of 12p with log2 ratio of 0.226, 0.356, and 0.58, respectively. (d, e) Karyotype and FISH results on cultured fibroblasts for patient 5. (d) Karyotype in R‐band illustrating the presence of supernumerary i(12) (p10) (red arrow). (e) FISH on metaphase spread confirming the presence of i(12p) (white arrow) using (12ptel27) probe in green and D12Z3 probe in red. (f) Interphase FISH of uncultured lymphocytes of patient 1 showing three red spots and two green spots. Red probe = D12Z3, Green probe = D2Z2. (g) FISH on nucleus of cultured lymphocytes of patient 2 showing four green spots for subtelomeric 12p probe and three red spots for D12Z3

Array Comparative Genomic Hybridization on DNA extracted from lymphocytes showed the presence of tetrasomy 12p in mosaic state in four patients out of five. The mosaicism percentages in aCGH analysis were 20 for patient 1 (log2 ratio value: 0.226), 28 for patient 2 (log2 ratio value: 0.356), and 50 for patient 5 (log2 ratio value: 0.58; Figure 1). aCGH in patient 3 revealed a distal gain of 12p with log2 ratio of 0.888 (85%) and proximal gain of short and long arm of chromosome 12 with log2 ratio of 0.281 (43%; Figure S1). aCGH result for patient 4 was normal; however, the PKS was confirmed using karyotyping and FISH on fibroblasts.

Results obtained from standard karyotypes, FISH for lymphocytes and/or fibroblasts for the five patients are summarized in Table 1. aCGH profiles for patients 1, 2 and 5 are presented in Figure 1 and for patient 3 in Figure S1.

**Table 1:** Summary of karyotypes, FISH, and aCGH results for the five PKS patients and the mosaic percentages

Patient	aCGH	Karyotype	Metaphase FISH	Interphase FISH	Tissue
1	Blood sample at the age of 21 months arr[hg19]12p13.33p11.1(163,593‐34,398,316)x2 ~ 3 20%	Blood sample at the age of 21 months mos 47,XY,+i(12)(p10)[1]/46,XY[49] 2%	ND	Blood sample at the age of 21 months nuc ish(D2Z2x2,D6Z1x2,D12Z3x3)[51]/ (D2Z2,D6Z1,D12Z3)x2[949] 5%	Peripheral blood
2	Blood sample at the age of 4 months arr[hg19]12p13.33p11.1(1–34,383,106)x2 ~ 4 28%	Blood sample at the age of 3 days 46,XY [15]	Blood sample at the age of 4 months ish12p13.3(12ptel27x2),12p11.1q11.1(D12Z3X2)[65] nuc ish(12ptel27x4,D12Z3X3)[7]/(12ptel27x2,D12Z3X2)[293] Total of 2%	ND	Peripheral blood
3	Blood sample at age of 4 month and 3 weeks arr[hg19]12p13.33p12.1(163,593–23,773,723)x3~4, 12p12.1q12(23,793,692–46,168,172)x2 ~ 3 85% for 12p13.33p12.1 43% for 12p12.1q12	Blood sample at age of 1 day mos 47,XX,der(12)t(12;12)(q12;p12.1)[3]/46,XX[57] 5%	Blood sample at age of 4 months and 3 weeks ish der(12)t(12;12)(q12;p12.1)(12ptel27++,D12Z3+,RP11‐478B9+)[10]/12p13.3(12ptel27x2),12p11.1q11.1(D12Z3x2),12q12(RP11‐478B9x2)[290] nuc ish(12ptelx4,D12Z3x3,RP11‐478B9x3)[55]/(12ptel,D12Z3,RP11‐478B9)x2[345] Total of 8%	ND	Peripheral blood
4	Normal result on peripheral blood at age of 18 months	Skin biopsy at age of 2 years and 4 months: mos 47, XY,+i(12)(p10)[4]/46,XY[16] 20%	Skin biopsy at age of 2 years at 4 months: ish12p11.1q11.1(D12Z3x3)[2/20] nuc ish(D12Z3x3)[10/100] Total of 10%	ND	Peripheral blood Skin biopsy
5	Blood sample at the age of 11 months arr[hg19]12p13.33p11.1(1–34,383,106)x4 50%	Blood sample at the age of 2 days: 46,XY[50]	Blood sample at the age of 2 days ish i(12p)(p10)(12ptel27++,D12Z3+)[3/140] nuc ish(12ptel27 x4,D12Z3 x3)[8/50] Total of 5.5% Blood sample at the age of 16 months: ish12(12ptel27,D12Z3)x2[450] nuc ish(12ptel27,D12Z3)x2	Blood sample at the age of 16 month: nuc ish(D12Z3x3)[64]/(D12Z3x2)[141] 31% nuc ish(12ptel27x4)[28]/(12ptel27x2)[74] 28%	Peripheral blood Skin biopsy
		Skin biopsy at age of 17 month: 47,XY,i(12)(p10)[12]	Skin biopsy at the age of 17 months: ish i(12)(p10)(12ptel27++,D12Z3+)[50] 100%		

Abbreviations: aCGH, array Comparative Genomic Hybridization; ND, not done; PKS, Pallister–Killian syndrome.

The clinical data for the five patients are summarized in Table 2.

**Table 2:** Comparison of phenotypes of our five PKS patients and review of previously reported postnatal patients

Phenotypes	Present patients	%	Reported patients *N* = 106 (Karaman et al., 2018) *N* = 15 (Wilkens et al., 2012) *N* = 59 (Blyth et al., 2015) *N* = 22 (Costa et al., 2015) *N* = 10	%
Dysmorphic features				
Frontotemporal balding	4/4	100	54/66	88
High forehead	5/5	100	35/66	53
Sparseness of eyebrows and/eyelashes	2/4	50	32/53	60
Upslanting palpebral fissures	1/4	25	31/44	70
Downslanting palpebral fissures	1/4	25	1/22	4.5
Telecanthus/epicanthus/ ptosis	2/5	50	21/80	24
Hypertelorism	5/5	100	16/25	61
Flat and broad nasal bridge	5/5	100	16/44	36
Short nose with upturned nares	4/4	100	23/59	40
Low‐set ears	5/5	100	14/59	24
Long philtrum/”Pallister lip”	5/5	100	28/59	47
Macroglossia	1/4	25	3/24	12
Short neck	4/6	66	33/34	77
Cleft palate	1/4	25	10/66	15
High‐arched palate	2/5	40	13/27	48
Accessory nipples	0/4	—	30/70	43
Short hands	1/5	20	17/35	47
Single palmar crease	2/5	40	11/56	17
Polydactyly	0/5	—	1/91	1
Clinodactyly/syndactyly	0/5	—	5/91	5
Large big toe	1/4	25	NA	
Lymphedema of hands or feet	0/5	—	23/55	42
Skin pigmentation	1/4	25	34/80	42
Neurological abnormalities				
Global developmental delay	5/5	100	34/47	72
Axial hypotonia	4/4	100	81/96	84
Intellectual disability	4/4	100	48/50	96
Epilepsy	3/5	60	65/94	65
Cerebral anomalies	3/4	75	33/56	59
Optic nerve hypoplasia	1/4	25	3/32	9
Hearing impairment	5/5	100	57/73	78
Strabismus	4/5	80	41/51	80
Growth				
IUGR	0/4	—	2/35	6
Microcephaly	0/3	—	22/60	36
Postnatal growth retardation	0/4	—	21/51	41
Short stature (SD)	0/4 [−0.5/−1.8] *SD*	—	21/51	41
Underweight (SD)	0/5 [−1.2/+1.5] *SD*	—	17/56	30
Cardiac defects	1/4	25	40/109	37
Respiratory abnormalities				
Lung anomalies	0/4	—	37/48	77
Laryngomalacia	3/4	75	NA	
Stridor	1/4	25	NA	
Gastrointestinal disorders				
CDH	0/4	—	5/77	6
GERD	3/4	75	18/48	36
Anal malformation	1/4	25	12/72	17
Umbilical hernia	1/4	25	15/90	17
Renal anomalies	2/4	50	5/56	9
Cryptorchidism	2/3	77	15/50	30
Musculoskeletal	1/5	20	17/58	29
Scoliosis	1/5	20	6/22	27

Abbreviation: NA, not available.

## 4 DISCUSSION

Pallister–Killian syndrome is a disorder associated with global developmental delay, multiple congenital anomalies, and mosaicism for isochromosome 12p. Our five patients exhibited typical phenotypes of PKS, including classic dysmorphic features, global developmental delay, receptive or conductive hearing impairment, strabismus, and gastroesophageal reflux disease ([GERD] Table 2; Wilkens et al., 2012). In our series, no patients manifested CDH since the latter associates with complications leading to fetal demise, or it could be diagnosed prenatally and subsequent a termination of pregnancy (Blyth et al., 2015). One patient demonstrated cardiac anomalies which are frequently observed in PKS patients. Epilepsy in PKS usually occurs in the first 4 years of life (Blyth et al., 2015; Wilkens et al., 2012). We observed epilepsy in three of five patients starting between 4 and 7 years; the other two patients are young (3 years and 4 and half years) and therefore a long‐term follow‐up will be needed in order to completely rule out the possibility  of manifesting epilepsy. One patient showed optic nerve hypoplasia which has rarely been reported in PKS (Blyth et al., 2015).

### 4.1 Cytogenetic techniques to detect the presence of isochromosome 12p

During the postnatal period, the conventional cytogenetic methods might not reveal the presence of mosaicism for isochromosome 12p on peripheral blood samples as shown in patients 2 and 5 (Conlin et al., 2012). Our data demonstrated different percentages of mosaic i(12p) in cultured and uncultured lymphocytes, suggesting that cultured lymphocytes might undergo a negative selection for marker chromosome (Pagon et al., 1979). Since conventional cytogenetic analysis requires actively dividing cells, it could make the detection of mosaic i(12p) unlikely (Reeser & Wenger, 1992). Therefore, the interphase FISH could slightly improve the detection level of mosaicism on peripheral blood since the process does not require cultured cells. However, FISH is a targeted technique. Since the PKS is a tissue‐limited mosaicism, the detection of i(12p) on skin biopsy by standard cytogenetic analysis is still the gold standard (Hodge et al., 2012). However, skin biopsy is an invasive procedure and the detection rate might decrease from the nonpigmented region (Harnden, 1960).

Identifying low level of mosaicism for chromosomal abnormalities by traditional cytogenetic techniques on peripheral blood cells is challenging. In comparison with cytogenetic analysis, aCGH provides results on extracted genomic DNA without cell cultures. Therefore, it might better reflect the value of mosaicism of cells (Ballif et al., 2006). Our results are similar to those previously reported as it illustrates that aCGH is superior to standard cytogenetic techniques and FISH in detecting mosaic i(12p) on peripheral blood (Ballif et al., 2006; Chen et al., 2014). We identified the mosaic i(12p) by aCGH first in three patients out of four which then facilitated finding the i(12p) by FISH and karyotype analysis. The fourth patient showed atypical tetrasomy 12p.

Our data supported the literature that aCGH on peripheral blood sample is highly sensitive in detecting mosaicism of isochromosome 12p (Blyth et al., 2015; Lee et al., 2017; Theisen et al., 2009; Table S1).

In our series, aCGH on peripheral blood identified a copy number gain of the short arm of chromosome 12 in four patients out of five, even with a low level of mosaicism of up to 20%. These results were confirmed by FISH. (Table 1). The age of detecting the tetrasomy 12p by aCGH in these four patients ranged between 4 months and 21 months. Nevertheless, aCGH failed to detect the i(12p) in one patient of our series (patient 4) at 18 months, which has been detected by standard cytogenetic method on skin biopsy. A similar normal result has been shown in a patient with clinical diagnosis of PKS who was tested using aCGH on peripheral blood at 2 years and 5 months. And therefore the skin biopsy was opted for aCGH at the same age which demonstrated the presence of tetrasomy 12p (Hodge et al., 2012). Another array‐based cytogenetic study (SNP array) of 15 patients aged between 8 days and 6 years and 9 months using peripheral blood sample and skin biopsy showed that the percentage of mosaic i(12p) cells on peripheral blood declined with the PKS patients ages, while the tetratomic cells were still quite stable overtime on skin biopsy using the SNP array (Conlin et al., 2012). In this study, the tetratomic cells on peripheral blood using SNP array were lost as early as 17 months old in one patient, while in other PKS patient these cells were still detectable at 6 years old using the SNP array on peripheral blood (Conlin et al., 2012). In our series, the oldest diagnosed patient by aCGH was 21 months old (patient 1) while the other three diagnosed patients were less than 1 year old. The tetratomic cells were undetectable using aCGH in patient 4 at 18 months. We really do not know when those tetrasomic cells exactly become undetectable on the blood (Conlin et al., 2012). Thus, it might be better to perform the aCGH as a first‐tier method on peripheral blood at early age, under 12 months, when PKS is clinically suspected to eliminate the need for undertaking skin biopsy at older age. However, in highly suspected PKS casses, older than 12 months, who have normal aCGH results on lymphocytes, a buccal smear and/or skin biopsy specimen might be opted for FISH, karyotypes and/or aCGH as a second‐tier test (Conlin et al., 2012; Hodge et al., 2012).

On the other hand, aCGH cannot determine the cause of the entire 12p gain, thus emphasizing the importance of confirming this result by FISH (Chen et al., 2014).

### 4.2 The 3rd patient (derivative 12)

To the best of our knowledge, this is the first reported patient of PKS resulting from an unbalanced translocation between the two chromosomes 12 homologous. The mechanism proposed is that a U‐loop exchange occurred between the short and the long arm of homologous chromosome 12 followed by nondisjunction errors through meiosis. Results in supernumerary derivative chromosome contain two copies of 12p13.33p12.1 (partial tetrasomy) and one copy of 12p12.1q12 (partial trisomy). The marker chromosome has been characterized by aCGH and the result has been confirmed by FISH. The patient has the classical phenotype of PKS. Nevertheless, she has neither diaphragmatic hernia nor cardiac defect. The patient is 3 years old and therefore could manifest seizures later on. Izumi et al. (2012) published a paper redefining a minimal critical region responsible for PKS patients that is completely included in the chromosomal abnormalities detected in this patient. The most frequent cause of PKS is the supernumerary i(12p). The mechanism suggested for i(12p) is nondisjunction errors through meiosis, followed by the centromeric misdivision. Other variants for PKS have been reported (Table S2; Lloveras et al., 2013; Yeung, Francis, Giouzeppos, & Amor, 2009).

## 5 CONCLUSION

PKS is tissue‐limited mosaicism due to tetrasomy 12p. Our cohort exhibits the typical phenotypes of PKS, including classic dysmorphic features, global developmental delay, epilepsy, hearing impairment, and strabismus. Here, we confirmed that aCGH on peripheral blood sample may be considered as a first‐tier method to use when the PKS is suspected especially at early age. Moreover, the use of aCGH on a blood sample might avoid the need for a skin biopsy. Finally, the characterization of unbalanced chromosomal aberration and marker chromosome is more efficient by aCGH, which provides high resolution.

## CONFLICT OF INTEREST

None declared.
