# Supplementary material for: Postnatal clinical phenotype of five patients with Pallister–Killian Syndrome (tetrasomy 12p): Interest of array CGH for diagnosis and review of the literature
Source: Mol Genet Genomic Med. 2019 Aug 27;7(10):e00939. doi: 10.1002/mgg3.939 (PMC6785526; doi:10.1002/mgg3.939)
Supplement: Supplementary file 1 [file MGG3-7-e00939-s001.docx]

**SUPPLEMENTARY INFORMATION**


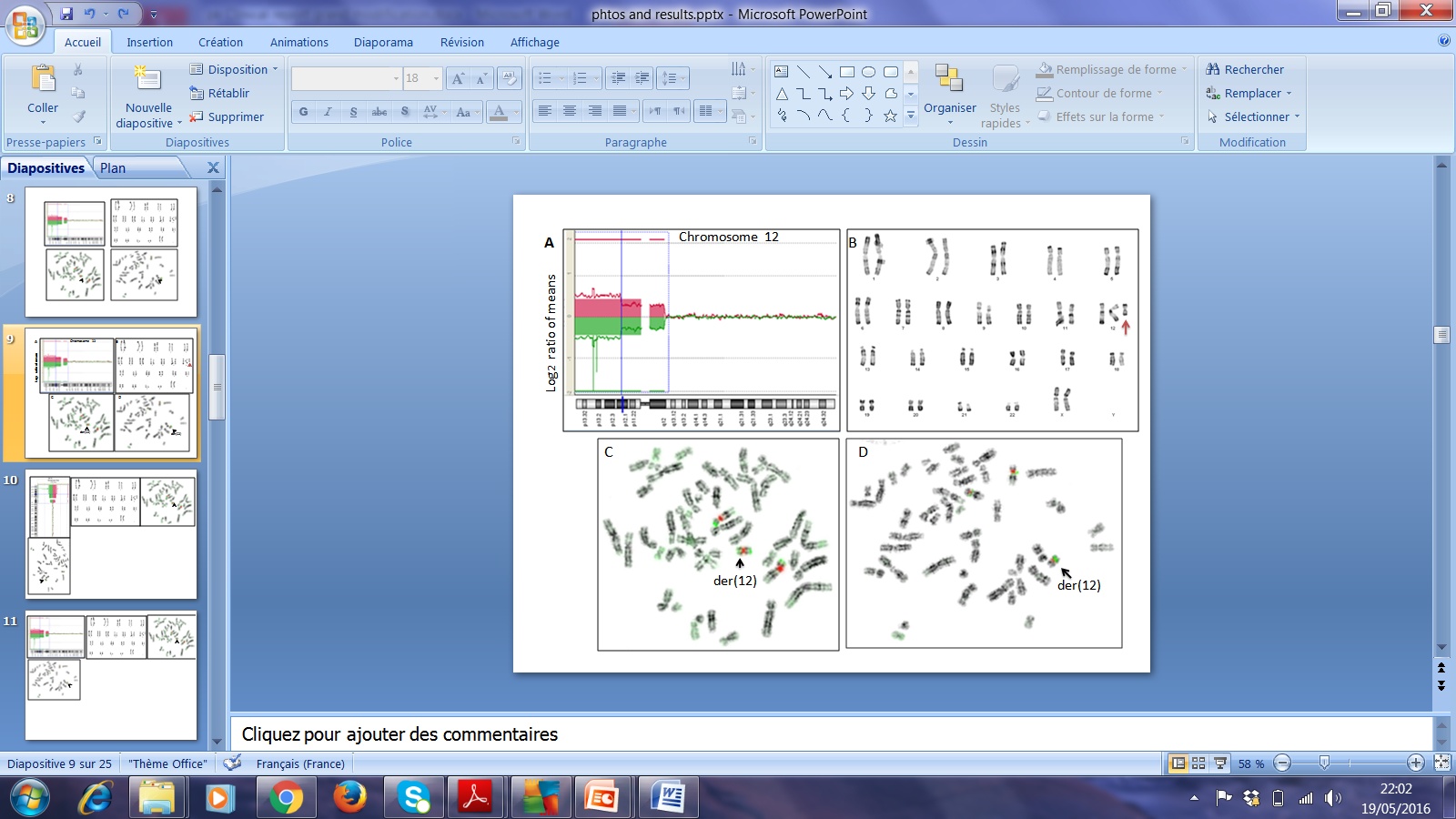


**Supplementary figure 1. aCGH, karyotype and FISH result for the third patient.** (A) aCGH profile of uncultured lymphocytes illustrating gain of the distal part of 12p with log2 ratio of 0.888 and a gain of proximal part of 12p and 12q with log2 ratio of 0.281. (B) Karyotype on lymphocytes indicating the presence of the supernumerary chromosome. (C-D) FISH on metaphase spreads on cultured lymphocytes. (C) Confirms the presence of supernumerary chromosome with 2 green signals for 12ptel27 probe and red signal for D12Z3 probe on the supernumerary chromosome. (D) Shows one green signal for PR11-478B9 probe along with red signal for D12Z3 probe on the supernumerary chromosome.

| **Supplementary table 1. Comparison of aCGH percentage for copy-number gain of 12p in our series and in the literature** | | | | | |
| --- | --- | --- | --- | --- | --- |
| **Reference** | **Age at the time of diagnosis by array based cytogenetic method** | **Number of cases** | **Array-type** | **Percentage of mosaicism** | **Specimen** |
| This study | 4 months-21 months | 5 | aCGH | 20 – 85 % in 4 cases | Peripheral blood |
| (Lee et al. 2017) | 9 months-  4 years and 10 months | 2 | aCGH | The percentage has not been specified | Peripheral blood |
| (Blyth et al. 2015) | 4 months-2 years | 3 | aCGH | 8.5 %  22.5 %  50% | Peripheral blood |
| (Chen et al. 2014)* | Prenatal at 20 WG.  TOP at 22 WG. | 1 | aCGH | 87 %  62.5 %  62.5 % | Amniocentesis  Cordocentesis  Skin biopsy |
| (Lloveras et al. 2013) | Prenatal at 16 WG | 1 | aCGH | 25% | Amniocentesis |
| (Theisen et al. 2009) | Postnatal/ Age was not mentioned | 7 | aCGH | Identified in all cases but the percentages have not been specified | - Peripheral blood in 7/7 cases  - Skin biopsy in 3/7 cases |
| (Conlin et al. 2012)* | 8 days-6 years and 9 months | 15 | SNP array | - 35% - 100% in 13/13 cases - 5%-75% in 6/13 cases | Skin biopsy  Peripheral blood |
| (Hodge et al. 2012) | 2 years and 5 months | 1 | aCGH | 0 % on peripheral blood  50 % on skin biopsy | Peripheral blood  Skin biopsy |
| (Ballif et al. 2006) | Postnatal/ Age was not mentioned | 1 | aCGH | 24%  51.5 % | Peripheral blood  Skin biopsy |
| *Mosaicism tetrasomy/hexasomy 12p.  TOP: Termination of pregnancy.  * In Conlin et al, a total of 15 patients were analyzed using SNP array among them, 11 had both blood and skin samples analyzed, two patients had only blood analyzed and two patients had only skin analyzed. | | | | | |

| **Supplementary table 2. Reported mechanism of tetrasomy 12p other than i(12p) in the literature** | | | | |
| --- | --- | --- | --- | --- |
| **Article** | **Age** | **Mechanism** | **Tissue** | **Technique** |
| Present study | Postnatal | Translocation 12;12 | Peripheral blood | aCGH  Karyotype  FISH |
| (Chen et al. 2014) | Prenatal  postnatal | Tetrasomy 12p/ trisomy12p | Amniocentesis  Cordocentesis  Skin biopsy | aCGH  Karyotype  FISH |
| (Lloveras et al. 2013) | Prenatal | Ring chromosome of 12p | Amniocentesis | Karyotype  FISH  aCGH |
| (Yeung et al. 2009) | Postnatal | Ring chromosome of 12p | Skin biopsy  Buccal smear | Karyotype  FISH  Interphase FISH |
| (Huang et al. 2007) | Postnatal | Partial tetrasomy 12p  (inverted duplication of  12pter-12p11.22) | Skin biopsy  Buccal smear | Karyotype  FISH  Multicolor-FISH  aCGH  Microsatellites for chromosome 12p |
| (Vermeesch et al. 2005) | Postnatal | Partial tetrasomy 12p  (inverted duplication of  12pter-12p13.31) | Peripheral blood | Karyotype  FISH  Multicolor-FISH  Centromere specific multicolor FISH  aCGH  Microsatellites for chromosome 12p |
| (Dufke et al. 2001) | Postnatal | Partial tetrasomy 12p  (inverted duplication of 12pter-12p12.3) | Peripheral blood  Skin biopsy | Karyotype  FISH  Microsatellites for chromosome 12p |
| (Los et al. 1995) | Prenatal | Tetrasomy 12p/ trisomy12p | Amniocentesis | Karyotype  FISH |

**References**

Ballif, Blake C., Emily A. Rorem, Kyle Sundin, Matt Lincicum, Shannon Gaskin, Justine Coppinger, Catherine D. Kashork, Lisa G. Shaffer, et Bassem A. Bejjani. 2006. « Detection of Low-Level Mosaicism by Array CGH in Routine Diagnostic Specimens ». *American Journal of Medical Genetics. Part A* 140 (24): 2757‑67. https://doi.org/10.1002/ajmg.a.31539.

Blyth, Moira, Viv Maloney, Sarah Beal, Morag Collinson, Shuwen Huang, John Crolla, I. Karen Temple, et Diana Baralle. 2015. « Pallister-Killian Syndrome: A Study of 22 British Patients ». *Journal of Medical Genetics* 52 (7): 454‑64. https://doi.org/10.1136/jmedgenet-2014-102877.

Chen, Chih-Ping, Cheng-Ran Peng, Schu-Rern Chern, Yu-Ling Kuo, Peih-Shan Wu, Dai-Dyi Town, Chen-Wen Pan, Chien-Wen Yang, et Wayseen Wang. 2014. « Interphase Fluorescence in Situ Hybridization Characterization of Mosaicism Using Uncultured Amniocytes and Cultured Stimulated Cord Blood Lymphocytes in Prenatally Detected Pallister-Killian Syndrome ». *Taiwanese Journal of Obstetrics & Gynecology* 53 (4): 566‑71. https://doi.org/10.1016/j.tjog.2014.09.004.

Dufke, A., C. Walczak, T. Liehr, H. Starke, V. Trifonov, N. Rubtsov, M. Schöning, H. Enders, et T. Eggermann. 2001. « Partial Tetrasomy 12pter-12p12.3 in a Girl with Pallister-Killian Syndrome: Extraordinary Finding of an Analphoid, Inverted Duplicated Marker ». *European Journal of Human Genetics: EJHG* 9 (8): 572‑76. https://doi.org/10.1038/sj.ejhg.5200673.

Hodge, Jennelle C., Rachael L. Hulshizer, Pam Seger, Angelique St Antoine, Jennifer Bair, et Salman Kirmani. 2012. « Array CGH on Unstimulated Blood Does Not Detect All Cases of Pallister-Killian Syndrome: A Skin Biopsy Should Remain the Diagnostic Gold Standard ». *American Journal of Medical Genetics. Part A* 158A (3): 669‑73. https://doi.org/10.1002/ajmg.a.35209.

Huang, X.-L., M. Isabel de Michelena, E. Leon, T. A. Maher, R. McClure, et A. Milunsky. 2007. « Pallister-Killian Syndrome: Tetrasomy of 12pter-->12p11.22 in a Boy with an Analphoid, Inverted Duplicated Marker Chromosome ». *Clinical Genetics* 72 (5): 434‑40. https://doi.org/10.1111/j.1399-0004.2007.00894.x.

Lee, Mi Na, Jiwon Lee, Hee Joon Yu, Jeehun Lee, et Sun Hee Kim. 2017. « Using Array-Based Comparative Genomic Hybridization to Diagnose Pallister-Killian Syndrome ». *Annals of Laboratory Medicine* 37 (1): 66‑70. https://doi.org/10.3343/alm.2017.37.1.66.

Lloveras, E., A. Canellas, V. Cirigliano, V. Català, C. Cerdan, et A. Plaja. 2013. « Supernumerary Ring Chromosome: An Etiology for Pallister-Killian Syndrome? » *Fetal Diagnosis and Therapy* 34 (3): 172‑75. https://doi.org/10.1159/000347049.

Los, F. J., D. Van Opstal, M. P. Schol, J. L. Gaillard, H. Brandenburg, A. M. Van Den Ouweland, et P. A. in ’t Veld. 1995. « Prenatal Diagnosis of Mosaic Tetrasomy 12p/Trisomy 12p by Fluorescent in Situ Hybridization in Amniotic Fluid Cells: A Case Report of Pallister-Killian Syndrome ». *Prenatal Diagnosis* 15 (12): 1155‑59.

Theisen, Aaron, Jill A. Rosenfeld, Sandra A. Farrell, Catharine J. Harris, Heather H. Wetzel, Beth A. Torchia, Bassem A. Bejjani, Blake C. Ballif, et Lisa G. Shaffer. 2009. « ACGH Detects Partial Tetrasomy of 12p in Blood from Pallister-Killian Syndrome Cases without Invasive Skin Biopsy ». *American Journal of Medical Genetics. Part A* 149A (5): 914‑18. https://doi.org/10.1002/ajmg.a.32767.

Vermeesch, Joris Robert, Cindy Melotte, Ivo Salden, Mariluce Riegel, Vladimir Trifnov, Anna Polityko, Natalia Rumyantseva, et al. 2005. « Tetrasomy 12pter-12p13.31 in a Girl with Partial Pallister-Killian Syndrome Phenotype ». *European Journal of Medical Genetics* 48 (3): 319‑27. https://doi.org/10.1016/j.ejmg.2005.04.018.

Yeung, Alison, David Francis, Olivia Giouzeppos, et David J. Amor. 2009. « Pallister-Killian Syndrome Caused by Mosaicism for a Supernumerary Ring Chromosome 12p ». *American Journal of Medical Genetics. Part A* 149A (3): 505‑9. https://doi.org/10.1002/ajmg.a.32664.
